# Association between Family History of Breast Cancer and Breast Density in Saudi Premenopausal Women Participating in Mammography Screening

**DOI:** 10.3390/clinpract14010013

**Published:** 2024-01-19

**Authors:** Ibrahem Hussain Kanbayti, Mayada A. Alzahrani, Yara O. Yeslam, Noora H. Habib, Ibrahim Hadadi, Yousef Almaimoni, Adnan Alahmadi, Ernest U. Ekpo

**Affiliations:** 1 Radiologic Sciences Department, Faculty of Applied Medical Sciences, King Abdulaziz University, Jeddah, Saudi Arabia; mayada.ab114@gmail.com (M.A.A.); yabas99@hotmail.com (Y.O.Y.); norahabib198@gmail.com (N.H.H.); yalmaimoni@kau.edu.sa (Y.A.); aaalahmadi@kau.edu.sa (A.A.); 2 Department of Radiological Sciences, College of Applied Medical Sciences, King Khalid University, Abha, Saudi Arabia; ihadadi@kku.edu.sa; 3 Medical Image Optimization and Perception Group (MIOPeG), Faculty of Medicine and Health, The University of Sydney, Campus C4 75 East Street, Sydney, NSW 2141, Australia; ernest.ekpo@sydney.edu.au

**Keywords:** mammographic density, family history of breast cancer, premenopausal women

## Abstract

Background: Mammographic density and family history of breast cancer (FHBC) are well-established independent factors affecting breast cancer risk; however, the association between these two risk factors in premenopausal-screened women remains unclear. The aim of this study is to investigate the relationship between mammographic density and FHBC among Saudi premenopausal-screened women. Methods: A total of 446 eligible participants were included in the study. Mammographic density was assessed qualitatively using the Breast Imaging Reporting and Data System (BIRADS 4th edition). Logistic regression models were built to investigate the relationship between mammographic density and FHBC. Results: Women with a family history of breast cancer demonstrated an 87% greater chance of having dense tissue than women without a family history of breast cancer (95% CI: 1.14–3.08; *p =* 0.01). Having a positive family history for breast cancer in mothers was significantly associated with dense tissue (adjusted odds ratio (OR): 5.6; 95% CI: 1.3–24.1; *p* = 0.02). Conclusion: Dense breast tissue in Saudi premenopausal women undergoing screening may be linked to FHBC. If this conclusion is replicated in larger studies, then breast cancer risk prediction models must carefully consider these breast cancer risk factors.

## 1. Introduction

Family history of breast cancer (FHBC) and high breast density are well-established breast cancer risk factors [1,2,3]. Women whose closest relatives had a history of breast cancer have a two-to-fourfold elevated risk of developing breast cancer [1]. Epidemiological studies also show that women with extensive amount of fibroglandular breast tissue, represented by brighter regions on a mammogram, have a two-to-four times higher risk of developing breast cancer [3,4]. Both mammographic density and FHBC have a heritable component and predispose women to breast cancer [5,6,7,8,9]. Therefore, it is plausible that FHBC may be associated with a woman’s mammographic density; however, published studies have reported discordant findings. While some studies have reported a relationship between FHBC and mammographic density [10,11,12,13], one study found no association [14].

Mammographic density and FHBC are determinants of breast cancer risk [1,2,3]. Mammographic density is associated with interval cancer and affects the early detection of breast cancer [15,16,17]. Therefore, it is increasingly important to investigate the association between mammographic density and FHBC. By understanding how FHBC and mammographic density are related, doctors can make more informed decisions about breast cancer screening that are tailored to women’s needs and risks.

There is limited evidence regarding the relationship between mammographic density and the degree of relative who has/had breast cancer [11,12,18]. Only two studies have sought to determine the relation between mammographic density and family history of breast cancer according to the person’s relationship to the family member with breast cancer [11,12]. These studies showed higher odds of dense breast tissue among women with family history of breast cancer in a sister alone. Since ethnic and geographic differences in breast density were documented among different populations [19,20,21], it is increasingly important to investigate the relationship between mammographic density and FHBC in the Saudi population. To our knowledge, no study has explored the relationship between these two determinants of breast cancer risk in a Saudi population. Furthermore, age and body mass index (BMI) have been shown to be negative confounders for mammographic density [22,23,24], and explain 20% and 30% of population differences in mammographic density [25]. Therefore, these factors must be considered when investigating the relationship between mammographic density and FHBC. However, these factors were not considered in the Iraqi population study [10].

To our knowledge, the literature on the association between mammographic density and FHBC were either based on a mixed population (premenopausal and postmenopausal women) or postmenopausal women [10,11,13,26]. Postmenopausal women have less dense tissues that might not allow us to sufficiently capture the relationship between FHBC and mammographic density [24].

To address the limitations mentioned above, this study aims to explore the association between mammographic density and FHBC among Saudi premenopausal women and the confounding effects of age and body mass index.

## 2. Materials and Methods

### 2.1. Study Population

**Figure 1:**
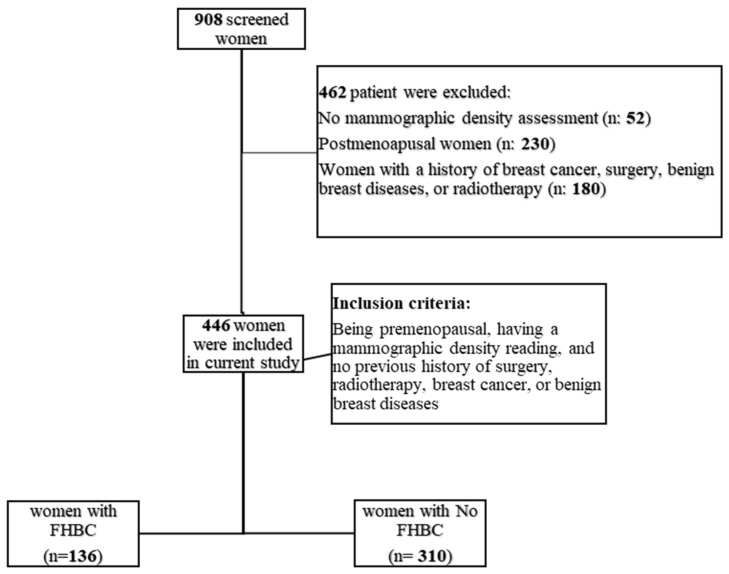
Flow chart of participants who met inclusion/exclusion criteria for the study population (FHBC: family history of breast cancer).

Between April 2012 and June 2017, 908 women were screened for breast cancer at Sheikh Mohammed Hussien AL-Amoudi Center of Excellence in Breast Cancer. Of the 908 individuals who participated, 446 were women who met the eligibility criteria for the current study. These criteria included being premenopausal, having a mammographic density reading, and no previous history of surgery, radiotherapy, breast cancer, or benign breast diseases. Postmenopausal women (*n* = 230), women without mammographic density assessment (*n* = 52), and women with a history of breast cancer, surgery, benign breast diseases, or radiotherapy (*n* = 180) were excluded from the study (see Figure 1).

### 2.2. Covariates

As part of the screening process, women were requested to complete a questionnaire providing information on their demographic profile (age at screening), anthropometric measurement (BMI), family history of breast cancer (status of family history, the degree of relative who had/has breast cancer), menopause status, and reproductive as well as hormonal background (parity, breast feeding, oral contraceptive administration).

### 2.3. Mammographic Density Assessment

Mammographic density was qualitatively assessed by a women’s imaging consultant (more than 25 years of experience). The Breast Imaging Reporting and Data System (BIRADS 4th edition scheme) was used to categorize breast density of women into BIRADS A (almost entirely fatty), BIRADS B (scattered fibroglandular tissue), BIRADS C (heterogeneously dense), and BIRADS D (extremely dense). To facilitate the interpretation of the study findings, this scale was collapsed into two categories: low breast density (BIRADS A and BIRADS B) and high breast density (BIRADS C and BIRADS D).

### 2.4. Statistical Analysis

Chi-squared test (X^2^) and Mann–Whitney U test were utilized to examine the differences between women with FHBC and women with no FHBC in terms of population characteristics, such as age at screening (continuous variable), menopause status (premenopausal, postmenopausal), BMI (continuous variable), parity (have children, have no children), education level, oral contraceptive pill administration (used, never used), and breast feeding (breastfed, never breastfed). Unadjusted logistic regression models were performed to investigate the relationship of FHBC (has FHBC, has no FHBC), and the degree of relative with breast cancer (FHBC in mother alone, FHBC in sister alone), with mammographic density. To determine whether the association between FHBC and mammographic density is influenced by age at screening and BMI, models were adjusted for these potential confounders. Odds ratios (ORs), as well as confidence intervals (95% CIs), were utilized to describe the analysis. All tests were two-sided, and the *p* value considered statistically significant if it was less than 0.05. IBM SPSS software, version 25 (IBM Crop., Armonk, NY, USA) was utilized to perform the analyses.

## 3. Results

**Figure 2:**
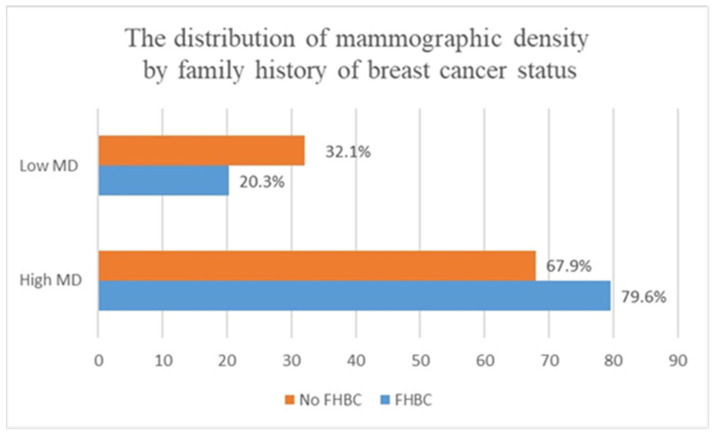
The distribution of mammographic density by FHBC (*p* value: 0.01); family history of breast cancer: FHBC; MD: mammographic density; low density: BIRADS A + BIRADS B; high density: BIRADS C + BIRADS D).

A total of 136 women with FHBC and 310 females without FHBC were included in the present study. Compared to women without FHBC, participants with FHBC were more likely to have a higher baseline mammographic density (79.6% vs. 67.9%) (see Figure 2). No significant differences were found between women with FHBC and women without FHBC in terms of body mass index, parity, age, breast feeding, and oral contraceptive pills administration (*p* value > 0.2) (see Table 1).

In the multivariable logistic regression analysis (see Table 2), women with FHBC had 87% higher odds of dense tissue compared with women with no FHBC (95% CI: 1.14–3.08; *p* = 0.01). Having a positive FHBC in mothers was significantly associated with dense tissue after adjusting for confounders (adjusted OR: 5.6; 95% CI: 1.3–24.1; *p*=0.02) (see Table 3). No significant heterogeneities were found between mammographic density and FHBC in sisters before or after adjustment for age and BMI (*p* values = 0.2) (see Table 4).

## 4. Discussion

The findings from the study show that FHBC is a significant determinant of mammographic density independent of age and BMI, and that the contribution of FHBC to breast density is significantly stronger among women with positive FHBC in their mother alone. The underlying processes and etiologic nature of the association between mammographic density and familial history of breast cancer remain uncertain. Nevertheless, certain potential mechanisms have been elucidated through research. It has been demonstrated that mutations in genes responsible for DNA repair and apoptosis are more prevalent in dense breast tissues and among women with a strong familial history of breast cancer [27,28]. In the event that DNA damage occurs in normal stromal breast tissue, a series of events involving DNA repair genes would be initiated, presumably suppressing the expression of CD36, a glycoprotein receptor that governs the stromal environment of the breast [29]. Ultimately, this would lead to an increase in the deposition of extracellular matrix and a reduction in lipid storage in normal breast tissue, thereby reflecting the heightened density observed in women with a familial history of breast cancer.

Our findings on the association between FHBC and mammographic density agree with previous studies that examined other ethnic populations [10,12,13]. For example, Han et al. studied the use of quantitative and qualitative mammographic density assessment methods, as well as adjusting for age and BMI, and reported a positive association between mammographic density and FHBC among American premenopausal women [12]. An Iraqi study of 750 women utilizing a visual mammographic density assessment method to measure breast density found that women with FHBC have higher BIRADS density scores [10]. Similarly, a large American study conducted by Ziv et al. showed that women with higher BIRADS have 70% higher odds of having a first-degree relative with breast cancer [13]. However, our work differs from the two latter studies in that it mainly includes premenopausal women and focuses on the Saudi population. Compared to postmenopausal women, premenopausal women with dense breast tissue are more at risk for developing breast cancer [30,31]. Additionally, premenopausal women have sufficient amounts of fibroglandular tissues that could reflect the actual association between mammographic density and FHBC. In contrast to our findings, Yang et al.’s study reported no relationship between mammographic density and FHBC [14]. However, this study differed from the present study in that it relied on very limited number of women with FHBC, which may explain the discordant findings of the studies.

A few studies have examined the relationship between mammographic density and the degree of relative with FHBC [11,12,18]. Crest et al.’s study, for example, found that women with positive FHBC in first-degree relatives have 17% higher odds of having dense breasts [11]. Findings from the Maskarinec et al. study likewise found that first-degree FHBC is associated with increased odds of having dense breast tissues (adjusted odds ratio: 15.16; 95% CI: 4.23–54.28) [18]. Recent findings from the study by Han et al. showed increased odds of 27% and 32% of having dense breast tissues among women with positive FHBC in mothers and sisters, respectively [12]. In the current study, the association between mammographic density and FHBC was significantly pronounced among women with positive FHBC in their mothers. The null findings for the relationship between mammographic density and FHBC in sisters in our work can be attributed to the limited number of women with positive FHBC in sisters.

Our findings provide support for the hypothesis that mammographic density and FHBC may share genetic components that potentially cause breast cancer. Women possessing a pathogenic BRCA1 or BRCA2 variant were found to be at an elevated susceptibility to breast cancer, wherein this risk appears to be more pronounced amongst individuals with a first-degree familial background [32]. It has also been shown that breast cancer and mammographic density share similar single nucleotide polymorphisms (SNPs), which indicate a common genetic predisposition [33,34]. Recently, the BRCA1 mutation was found to be associated with an increased amount of collagen protein, a major breast tissue component of extracellular matrix that affects mammographic density [35].

Currently, in the absence of genomic information, the screening of Saudi women with a high risk of breast cancer is guided by FHBC. However, Saudi women’s knowledge of their FHBC has been reported to be poor [36]. Additionally, this screening strategy is unlikely to be effective as not all patients with FHBC will develop the disease. Therefore, the screening of women with FHBC should consider mammographic density data. This would allow for the earlier detection of cancer and a decrease in mortality rates. As there are currently no established local guidelines in Saudi Arabia for the screening of women with a high risk profile of breast cancer, our findings propose that women with a strong family history of breast cancer (first-degree relative) or dense breast tissue should undergo screening using magnetic resonance imaging (MRI) rather than mammography. When compared to MRI, mammography has lower sensitivity in the detection of cancers in dense breast tissues, which could increase the risk of missing cancers.

To the best of our knowledge, this study is the first to examine the relationship between mammographic density and FHBC among Saudi women. To date, much of the mammographic density studies conducted in the Saudi population focus on the relationship between mammographic density and breast cancer risk [37,38]. Thus, this study may provide preliminary data to guide the risk assessment and screening of Saudi premenopausal women. However, the current study has some limitations. The study’s small sample size restricts the generalizability of its findings to the overall population. This limitation also hindered the researchers’ ability to conclusively demonstrate a relationship between mammographic density and FHBC in sisters alone. Additionally, mammographic density was measured by a single observer using the qualitative assessment method (BIRADS). This subjective assessment method has been shown to have a wider inter- and intra-observer variability ranging from 0.37 to 0.91, suggesting its limited reproducibility in the current study [39]. Therefore, further larger studies considering highly reproducible quantitative mammographic density assessment methods are needed to confirm our study findings.

In conclusion, mammographic density may be strongly associated with FHBC among Saudi premenopausal screened women. Confirming this finding in larger studies could highlight the potential genetic link between mammographic density and FHBC, as well as help make informed decisions about screening frequency and pathways for premenopausal Saudi women with a family history of breast cancer.

## Figures and Tables

**Table 1:** Population characteristics by family history of breast cancer.

Population Characteristics		FHBC (*n* = 136)	No FHBC (*n* = 310)	*p* Value
BMI median (IQR)		28.44 (25.53–32.13)	28.51 (25.43–32.35)	0.8
Parity	Has children *n* (%)	121 (89.60)	277 (90.22)	0.84
	Has no children *n* (%)	14 (13.37)	30 (9.77)	
	Missing *n* (%)	1 (0.73)	3 (0.96)	
Breast feeding	Breastfed *n* (%)	114 (84.4)	253 (82.9)	0.6
	Never breastfed *n* (%)	21 (15.5)	52 (17)	
	Missing *n* (%)	1 (0.73)	5 (1.61)	
Age median (IQR)		44 (42–47)	44 (41–46)	0.1
OCP administration	Use *n* (%)	78 (57.7)	156 (551.3)	0.2
	Never use *n* (%)	57 (42.2)	148 (48.6)	
	Missing *n* (%)	1 (0.73)	6 (1.93)	

Abbreviations: family history of breast cancer: FHBC; BMI: body mass index; OCP: oral contraceptive pill; *n*: number of women.

**Table 2:** Odds ratios of high mammographic density in relation to family history of breast cancer.

Presence of FHBC	Presence of High Mammographic Density Crude OR (95% CI)	Presence of High Mammographic Density Adjusted OR (95% CI) ^a^
Women without FHBC (*n* = 310)	1 (Reference)	1 (Reference)
Women with FHBC (*n* = 136)	1.84 (1.12–3.02), *p* = 0.01	1.87 (1.14–3.08), *p* = 0.01

Abbreviations: OR: odds ratio; (95% CI): 95% confidence interval; FHBC: family history of breast cancer. ^a^: The model was adjusted for both age at screening (continuous variable), and BMI (continuous variable). *n*: number of women.

**Table 3:** Odds ratios of high mammographic density in relation to family history of breast cancer in the mother alone.

Presence of FHBC	Presence of High Mammographic Density Crude OR (95% CI)	Presence of High Mammographic Density Adjusted OR (95% CI) ^a^
No (*n* = 423)	1 (Reference)	1 (Reference)
Yes (*n* = 28)	5.5 (1.29–23.6), *p =* 0.02	5.6 (1.3–24.1), *p* = 0.02

Abbreviations: OR: odds ratio; (95% CI): 95% confidence interval; FHBC: family history of breast cancer. ^a^: The model was adjusted for both age at screening (continuous variable), and BMI (continuous variable). *n*: number of women.

**Table 4:** Odds ratios of high mammographic density in relation to family history of breast cancer in a sister alone.

FHBC (Sister)	Presence of High Mammographic Density Crude OR (95% CI)	Presence of High Mammographic Density Adjusted OR (95% CI) ^a^
No (*n* = 438)	1 (Reference)	1 (Reference)
Yes (*n* = 13)	2.31 (0.5–10.5), *p =* 0.27	2.34 (0.51–10.75), *p =* 0.27

Abbreviations: OR: odds ratio; (95% CI): 95% confidence interval; FHBC: family history of breast cancer. ^a^: The model was adjusted for both age at screening (continuous variable), and BMI (continuous variable). *n*: number of women.

## Data Availability

The data presented in this study are available on request from the corresponding author.
